# BisoGenet: a new tool for gene network building, visualization and analysis

**DOI:** 10.1186/1471-2105-11-91

**Published:** 2010-02-17

**Authors:** Alexander Martin, Maria E Ochagavia, Laya C Rabasa, Jamilet Miranda, Jorge Fernandez-de-Cossio, Ricardo Bringas

**Affiliations:** 1 Center for Genetic Engineering and Biotechnology, PO BOX 6162, Havana, Cuba

## Abstract

**Background:**

The increasing availability and diversity of omics data in the post-genomic era offers new perspectives in most areas of biomedical research. Graph-based biological networks models capture the topology of the functional relationships between molecular entities such as gene, protein and small compounds and provide a suitable framework for integrating and analyzing omics-data. The development of software tools capable of integrating data from different sources and to provide flexible methods to reconstruct, represent and analyze topological networks is an active field of research in bioinformatics.

**Results:**

BisoGenet is a multi-tier application for visualization and analysis of biomolecular relationships. The system consists of three tiers. In the data tier, an in-house database stores genomics information, protein-protein interactions, protein-DNA interactions, gene ontology and metabolic pathways. In the middle tier, a global network is created at server startup, representing the whole data on bioentities and their relationships retrieved from the database. The client tier is a Cytoscape plugin, which manages user input, communication with the Web Service, visualization and analysis of the resulting network.

**Conclusion:**

BisoGenet is able to build and visualize biological networks in a fast and user-friendly manner. A feature of Bisogenet is the possibility to include coding relations to distinguish between genes and their products. This feature could be instrumental to achieve a finer grain representation of the bioentities and their relationships. The client application includes network analysis tools and interactive network expansion capabilities. In addition, an option is provided to allow other networks to be converted to BisoGenet. This feature facilitates the integration of our software with other tools available in the Cytoscape platform. BisoGenet is available at http://bio.cigb.edu.cu/bisogenet-cytoscape/.

## Background

Network representation of relationships among biomolecules is an intensive field of research of *in silico *System Biology. New models for data integration, standard specifications for data exchange and the development of new tools for data visualization and analysis are crucial and represent one of the most challenging tasks for bioinformaticians.

Data repositories such as NCBI's Entrez Gene[1] and Ensembl[2] maintain annotation on whole genomes, including sequences, gene location, transcripts, classification and links to several external databases. Data retrieved from high-throughput experiments and literature are available from several databases, such as, DIP[3], BIND[4], HPRD[5], BioGRID[6], MINT[7] and Intact[8], which represent the major repositories of protein-protein interacions from multiple organisms. On the other hand databases like KEGG[9], Reactome[10], BioCyc[11], NCI Nature PID[12] and others provide information on both metabolic and signaling pathways. These databanks can be seen as repositories of biological entities and their functional relations. As the amount of biological data increase, software tools able to visualize biological-meaningful abstract representations of these data at different levels of details are valuable to biologists.

Graph-based model has shown to be a convenient model for representing the global picture of protein-protein interactions, transcription regulation, metabolic data and gene co-expression. In this model, bio-entities are represented as nodes in a graph, and functional relations (protein-protein interactions, transcription regulation and others) are represented as edges connecting the corresponding bio-entities. The particular properties of the bio-entities, and theirs functional relations are stored as node's and edge's attributes, respectively. In this way, in such abstract representation, the end-user can assess some of the most prominent features of the biological entities. However, many biological processes are characterized by more complex multiple relationships which are not compatible with graph representations. The use of hypergraphs may overcome such limitations. For an introduction on Hypergraphs and cellular networks see [13].

Several tools as Cytoscape[14,15], VisANT[16], Osprey[17] and Biological Networks[18], have being developed for reconstruction and visualization of networks of biological entities, for reviews see Pavlopoulos et al. [18] and Suderman et al[19,20]. Cytoscape is one of the most widespread software platforms for visualizing and integrating network data. It allows incorporating extra functionality due to flexible plug-in architecture. There are several plugins available for Cytoscape[21]. These plugins cover different functionalities such as: network inference, network analysis, functional enrichment and retrieving of network properties from external sources. Currently network building capabilities from remote data sources is provided by tools like Pathway Commons [22], Intact[23] web services clients and also trough MIMI [24] and APID2Net [25] plugins. Other tools handling biological networks have been also developed. BiNoM [26] is a Cytoscape plugin that is able to import network in multiple systems biology formats and carry out network structure analysis. CellDesigner [27] is a software suite that feature a friendly user interfaces for building gene-regulatory and biochemical models.

In most of network building tools that generate networks from database stored information, nodes represent genes and their protein products without distinguishing between them. However, with the increasing amount of information on microRNA genes and their targets, different gene isoforms and their specificity for tissue[28] and involvement in diseases[29], a need for independent visualization of genes and their products is becoming apparent. For example, representing an isoform-specific protein-protein interaction or different microRNAs coded by the same gene and targeting different mRNA will provide a better resolution for System Biology based research.

In this work we present BisoGenet a client-server based application for creating, visualizing and analyzing biological networks. This application relays on the biological information provided by SysBiomics, an in-house database integrating a wide range of omics information from multiple public data sources. BisoGenet client is designed to work as a Cytoscape plugin, featuring an easy to use interface for querying the server along with graph topology analysis and visualization options for easing the interpretation process.

## Implementation

**Figure 1:**
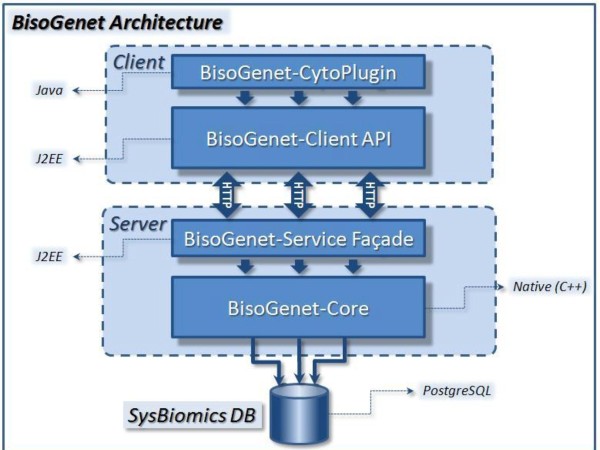
**BisoGenet general system architecture**. At the client tier a Plugin wrapper component provides a user interface for setting input options, sending request to the server and showing the results. This component is supported on BisoGenet Client API, which provides programmatic access to the Server and implements some functionality for managing the results. At the middle tier, the BisoGenet Service Façade, a J2EE based component, exposes the functionalities contained in the Core component through the web service technology. This Core component is implemented in C++. At the data tier SysBiomics, a PostgreSQL-managed database, integrates information on genes, proteins, protein-protein and protein-DNA interactions, gene ontologies and metabolic pathways from multiple sources.

BisoGenet is a client-server based application designed according to a multitier architecture. Three main independent deployable units compose this application: the data, the server and the client subsystems (figure 1). The data subsystem is a PostgreSQL relational database that integrates biological information from multiple sources and provides the information required by the server for building networks. The front-end client of BisoGenet is implemented as a Cytoscape plugin. This component provides visualization and graph topology analysis capabilities.

### The Data subsystem

BisoGenet's main functionality is focused on the construction of networks. Currently, our network model is based on genes, proteins and functional relationships between them such as protein-protein, protein-dna regulatory interactions and gene-protein coding relationship. Our database SysBiomics integrates heterogeneous data from multiple public domain datasets into a single and homogeneous repository. The database design reflects the nature of the data it contains. Biological entities such as genes, transcripts and proteins and their relationships define, to a large extent, the database structure. SysBiomics is supported on the open source PostgreSQL database manager, running on Linux. Access to SysBiomics data is provided through stored procedures, mostly implemented in pg/plsql leading to a performance boost of their execution.

In SysBiomics database population phase, gene data such as chromosome localization and exon composition of each of the splicing variants are imported from Entrez Gene [1] and NCBI Map Viewer [30]. Main protein information is provided by the major protein universal resource Uniprot[31]. Protein-protein and protein-DNA interactions information is taken from the Database of Interacting Proteins DIP[3], BIND[4], the Human Protein Reference Database HPRD[5], the Molecular INTeraction database MINT[7], Intact[8] and BioGrid[6]. Information on genes/proteins molecular function, biological processes and cellular component is imported from the Gene Ontology project [32]; while information on biochemical pathways is taken from KEGG [33]. Additional information includes links to databases OMIM[34], Unigene[35], PDB, Refseq[36], PFAM[37] and Pubmed[38].

In order to integrate all this data, SysBiomics creates an identifier translation table. This table maps common unambiguous gene and protein identifiers from EntrezGene, RefSeq, UniGene, GenBank and Uniprot into a unique internal identifier. All genes and protein poses at least one unambiguous identifier. The main source of ambiguity was gene aliases. There were a total of 5365 genes human genes with at least one redundant alias. For example, VH was an alias found in 36 different genes while GPCR was associated to 15. All this ambiguous identifiers were discarded in the identification process.

The data integration process gets gene/protein information from external sources and stores it in terms of a SysBiomics's unique identifier. The translation table includes different type of the most commonly used identifiers (see table 1 for a full list) which are used for the identification of the input list and for the mapping different data sources. The update of SysBiomics is performed quarterly with the more recent versions of the source databases.

**Table 1:** Types of identified supported by BisoGenet

Identifier source	Type	Example*
Entrez Gene official symbol	Gene	EGF

Entrez GeneId	Gene	1950

Entrez Gene RefSeq accession	Gene	NM_001963

Entrez Gene RefSeq Protein Id	Gene/Protein	NP_001954

Entrez Gene Alias	Gene	URG, HOMG4

Unigene Cluster ID	Gene	Hs.419815

GenBank Accession	Gene	AK299306
		AY548762
		BC093731
		BC113461
		X04571
		J02548

GenBank Protein Accession	Gene	EAX06257
		BAG61319
		AAS83395
		AAH93731
		AAI13462
		CAA28240
		AAA72173

Uniprot Id	Protein	EGF_HUMAN

Uniprot accession	Protein	P01133

Uniprot Secondary Accession	Protein	Q52LZ6

* EGF gene and its protein products IDs are given as an example

### The BisoGenet Server

The server subsystem (middle tier) provides the functionality for building networks. At server startup a single instance of a supergraph is created from the data contained in the SysBiomics. The supergraph is shared by all processing threads. This structure allows converting network construction queries into graph-based search operations. All genes and proteins of SysBiomics are represented by nodes in this graph. Genes are connected to the proteins they code for by an edge representing a coding relation. Each protein is connected to those proteins it interacts with by an edge representing a protein-protein interaction. And finally each protein is connected to genes it interact with, this edge represent a Protein-DNA interaction that occurs between the protein and a DNA sequence contained in the gene promoter.

The network building process consists of three steps: first, with the assistance of SysBiomics services each identifier from the input list is internally mapped to nodes in the supergraph (seed nodes). The mapped nodes represent the initial seed. In a second step, the network is expanded by interconnecting the seed nodes and adding nodes and edges from the super-graph, according to the source selection and expansion criteria stated in the query. In a third stage, certain information on the genes/proteins and the functional relations represented in the expanded graph are expressed in XML format. Finally this result is compressed and sent to the client.

The BisoGenet server was developed using J2EE technologies. However, due to performance and memory use optimization concerns, the core functionality of the service was implemented in C++ and built into native code. This server functionality was exposed through the wide-spread, platform-independent web services technology, using the Apache Axis Web service framework.

### The BisoGenet Client

The BisoGenet client is a Java desktop application designed as a Cytoscape's plugin. This application provides a user friendly interface presenting network construction options in an intuitive manner. Options specified by users are internally translated into query parameters and sent to the server on request. The server response is transformed into a graph and displayed on a Cytoscape's window according to a custom visual style. This client-server interchange is supported on the SOAP standard web service communication protocol over HTTP.

Unlike typical three tier applications, where the client job is almost restricted to visualization tasks, BisoGenet make use of client host processing power and run network analysis tools locally. These functionalities include: finding shortest paths between nodes, finding equivalent sets of nodes and calculating topological properties as the node degree and the cluster coefficient.

Once installed, Cytoscape add Bisogenet as an option in its Plugin menu. Menu items available in the BisoGenet plugin are "Create New Network", "Expand Current network", "Convert current network", "Find shortest paths", "Show network Statistics" and "Find equivalent nodes".

### The input

**Figure 2:**
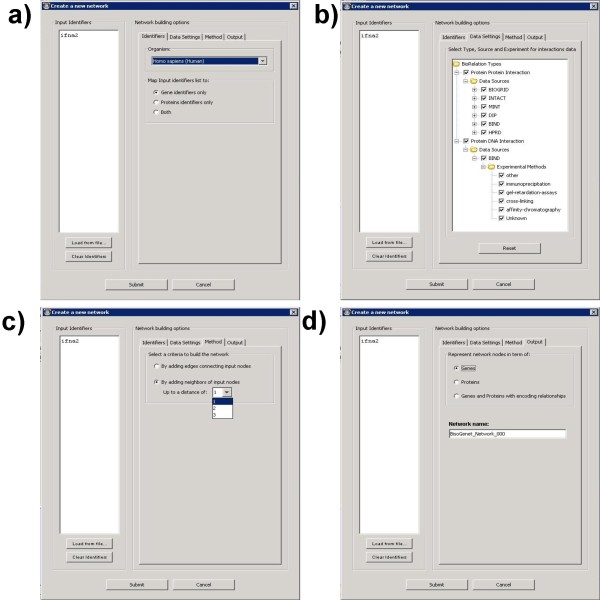
**BisoGenet Client input options**. Screenshots of Bisogenet input options. The text box on the left enables introducing a list of identifiers. a) Identifiers tab: a combobox allows choosing the organism to which the query genes/proteins belong to and the type of biological entities to be identified: genes only, proteins only or both. b) Data Settings: a tree component enables choosing data sources and the type of experimental methods to be considered. c) Method tab: it gives two alternatives for network building, first to build a network considering as nodes only those gene/proteins identified from the input list and second to build a network that includes in addition to those identified from the input list, neighbors located up to a distances of N edges (N is defined by the user). d) Output tab: It allows the user to choose network representation in terms of genes, proteins or both with coding relations.

The first step for building a new network is to define a seed set of gene or proteins. The user must provide a list of identifiers for the initial set of networks nodes and define input parameters. Figure 2 shows the four different tabs containing the input options required for building a BisoGenet network. First the user must define the organism the input data belongs to (figure 2.a), the data sources to be considered down to the level of the type of the experiment (figure 2.b), the distance from the input set to add new nodes to the network (figure 2.c) and the preferred representation for the out put (figure 2.d). A distinct feature of BisoGenet is the possibility to visualize relations derived from the central dogma of molecular Biology, the coding of a protein by a gene. In this case it is represented by a dashed line arrow as an edges directed from a gene to the proteins it codes for.

With the aim to cover a wide range of most commonly used identifiers and make the identification process as easy as possible for the final user, we studied the sources of data feeding Sysbiomics database and the set of identifiers most commonly used for people involved in Genomics and Proteomics research. From this analysis we choose the identifiers listed in table 1. As part of the analysis we look for possible cross links between different types of identifiers. We found that only in the case of "Entrez Gene Alias" some ids are common to more than one gene or are the same as one "Entrez Gene Symbol". We excluded those cases, this way in the identification process the user can provide a list of identifiers of types listed in table 1 and they will be unambiguously identified.

### Expanding a Network

Expanding an existing network is one of the capabilities provided by the client. Selecting a subset of nodes from an existing BisoGenet network and defining a new or the same set of input parameters it is possible to expand the current network.

### Network Statistics

Analytical network topology features were supported on freely available Java software libraries JUNG[39] and JFreeChart[40]. As part of JUNG library we also make use of CERN Colt Open Source Libraries for High Performance Scientific and Technical Computing in Java [41]. The options include network Statistics on node degree and cluster coefficient, Identification of equivalent nodes, or nodes with the same set of neighbors, and an option for finding the shortest path between all possible pairs of selected nodes.

### Converting to BisoGenet Network

This option is intended to incorporate BisoGenet functionality for networks generated by others software or imported from different sources. The conversion is possible if the external network use as node name some of the identifiers supported by BisoGenet.

## Results

BisoGenet was designed to assess the prominence of functional relations among sets of gene or proteins derived from Proteomics or Genomics experiments. Providing a list of identifiers, choosing the kind of relations to be included and choosing a selection criterion to add nodes to the network, the end user will easily and quickly obtain a network of functional related nodes. Node information available includes protein/gene description, GO terms and KEGG pathways with the corresponding links to external databases. Edges information includes the sources supporting the relations between the two connected nodes with links to the database web site, the type of the experimental method used as provided by the sources and the Pubmed references supporting the relation.

### Creating and expanding a network

**Figure 3:**
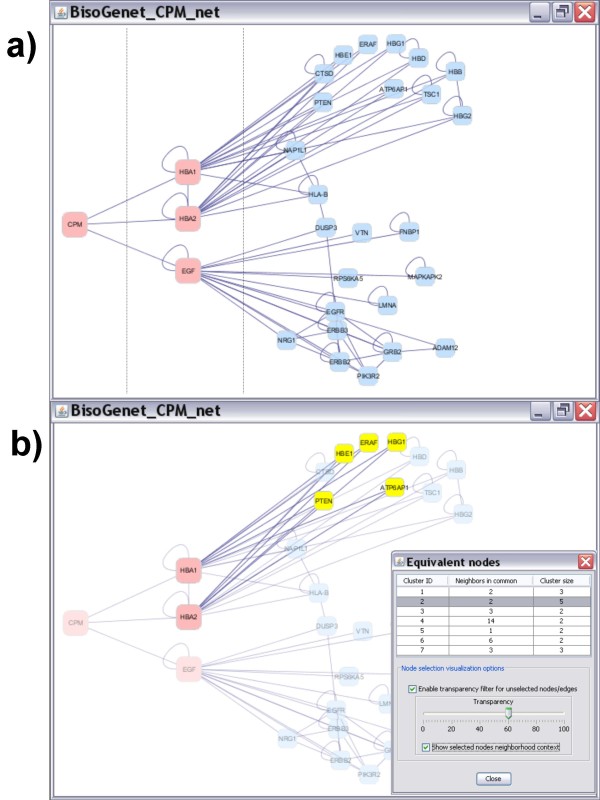
**Creating, expanding and analyzing a BisoGenet network**. a) Process of creating and expanding a network. The dashed lines divide the input node from the results; first, of creating a network and second of expanding it. First, CPM identifier is given by the user and a network is created by choosing to include neighbours up to a distance of 1 edge. Next the resulting network is expanded by choosing the added nodes and applying the same method as in first step. b) An example of the use of BisoGenet option for finding equivalent nodes or nodes with the same set of neighbors. A list of set of equivalents nodes is displayed with the number of equivalences and nodes in the set. Components of each set can be highlighted in the network by placing the cursor on it, additionally a transparency filter can be applied and the set of common nodes can be also highlighted.

An example of the network creation and expansion is illustrated in Figure 3. Introducing the gene name CPM as input and choosing to add neighbors nodes, genes HBA1, HBA2 and EGF are added and a network consisting of the 4 nodes (in red) is created. Next, nodes HBA1, HBA2 and EGF are selected and the network is further expanded.

### Analyzing a network

In figure 3.b it is illustrated one of Bisogenet network analysis tools "Find sets of equivalent nodes". Choosing this option a list of equivalent nodes, or nodes with the same set of neighbors is shown. Member nodes of a desired equivalent set can be highlighted in the network by clicking on that set on the list and selecting the transparency filter option. In the example in figure 3.b five equivalent nodes are shown, all of them interacting with both HBA1 and HBA2. In equivalent sets, functionally related genes are frequently found. Hence, when a protein of unknown function is found in a set of equivalent nodes and the rest of nodes in the set share common functions, those functions can be, in principle, extrapolated to the first. Also, the grouping of equivalent nodes may contribute to simplify the visualization of a complex networks. Two additional options "Find shortest path" and "Show network Statistics" share similar visualization options.

### Visualizing coding relations

**Figure 4:**
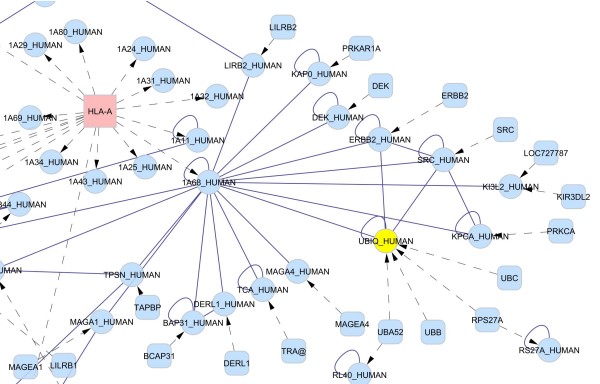
**Representing coding relations**. Partial view of a network created with gene name "hla-a" as input and choosing to add node up to a distance of one. The input node HLA-A, in red, represents a gene with multiple splice variants. The node UBIQ_HUMAN, in yellow, represent a protein coded by multiple genes. The output was chosen to include coding relation. Dashed lines with arrows represent coding relations directed from the gene node to protein nodes coded by each of the splice variants. BisoGenet assigns a distance of zero to coding relations. Solid lines represent protein-protein interaction networks.

One particular feature of BisoGenet is its capability to represent coding relations. In similar applications, when gene or protein networks are represented, networks node are usually treated as gene and/or protein indistinctly, regulatory and physical interactions are represented directed to a node that actually represent the gene and its products, proteins. Incorporating coding relations it is possible to distinguish between a gene and its protein products. Transcriptional regulatory relations can be directed from a protein (trancscription factor) to the genes its regulates. In the case of differential splicing it is possible to represent all variants of amino acid peptides coded by a gene. Protein-protein interactions specific to just one spliced variant of a gene can be also represented. In figure 4 we show an example of a network that includes coding relations. The network was generated using as input HLA-A, a gene with multiple splice variants. BisoGenet considers coding relations as of distance zero, so protein-protein interactions of HLA-A gene products are shown. For example, the splice variant product with more interactions reported is 1A02_HUMAN protein. Although, at present, it is difficult to imagine that the protein-protein interaction data are solved up to the level of each different gene product, providing these capabilities could be valuable for researchers dealing with disease- or tissue-specific splice variants. Also it allows visualizing cases of a protein being code for several genes, as is the case of protein UBIQ_HUMAN in figure 4.

## Conclusions

BisoGenet is a new tool for network building, visualization and analysis. One of its distinct features is the possibility of representing coding relations. Providing this capability it is possible to represent multiple isoforms of a gene as results of alternative splicing or the coding relations of two paralogous genes coding the same protein. With the increasing availability of information on disease-related [42] and tissue-specific [28] alternative splicing it is desirable to distinguish between different gene isoforms. On the other hand, the amount of regulatory information available is also increasing, like transcription factor-gene regulation derived from ChIP-chip and ChIP-seq studies and microRNA-gene silencing relations. One single gene can code for several microRNAs, each one targeting mRNAs transcribed by different genes. So taking all this together, incorporating coding relations is a desirable requirement for the development of more comprehensive System Biology oriented tools.

Future development of BisoGenet will focus on incorporating metabolic pathway visualization capabilities and new graph based algorithms for adding nodes to the networks. We also plan to add microRNA-gene silencing relations and new network analysis tools.

BisoGenet network visualization and analysis tool is freely available as a CytoScape plugin at http://bio.cigb.edu.cu/bisogenet-cytoscape/together with a user manual and installation instructions.

## Availability and requirements

Project name: BisoGenet

Project home page: http://bio.cigb.edu.cu/bisogenet

Operating system(s): Platform independent

Programming language: Java

Other requirements: Java 1.5 or higher, Cytoscape 2.6 or higher

License: free

Any restrictions to use by non-academics: no

## Authors' contributions

AM is the main programmer of the project, wrote the server layer code and part of the client code. MEO conceived the client component and wrote the initial version of its code, contribute to the code for populating the database. LCR contributed to the design of the database, implemented its first version and wrote the code for populating database and performed database updates. JM contributed to the database design and testing, wrote the latest addition to the database populating code and performed database updates. JFC contributed with ideas and advices to the project. RB conceived and directed the project. All authors contributed to database design. AM, MEO and RB drafted the manuscript and all the authors contributed to its final writing and approved the final manuscript.
